# The role of the amygdala during emotional processing in Huntington's disease: From pre-manifest to late stage disease

**DOI:** 10.1016/j.neuropsychologia.2015.02.017

**Published:** 2015-04

**Authors:** Sarah L. Mason, Jiaxiang Zhang, Faye Begeti, Natalie Valle Guzman, Alpar S. Lazar, James B. Rowe, Roger A. Barker, Adam Hampshire

**Affiliations:** a John Van Geest Centre for Brain Repair, University of Cambridge, UK; b Department of Clinical Neuroscience, University of Cambridge, UK; c MRC Cognition and Brian Sciences Unit, University of Cambridge, UK; d Division of Brain Sciences, Imperial College London, UK

**Keywords:** fMRI, Theory of mind, Amygdala, Effective connectivity, Reading the mind in the eyes

## Abstract

**Background:**

Deficits in emotional processing can be detected in the pre-manifest stage of Huntington's disease and negative emotion recognition has been identified as a predictor of clinical diagnosis. The underlying neuropathological correlates of such deficits are typically established using correlative structural MRI studies. This approach does not take into consideration the impact of disruption to the complex interactions between multiple brain circuits on emotional processing. Therefore, exploration of the neural substrates of emotional processing in pre-manifest HD using fMRI connectivity analysis may be a useful way of evaluating the way brain regions interrelate in the period prior to diagnosis.

**Methods:**

We investigated the impact of predicted time to disease onset on brain activation when participants were exposed to pictures of faces with angry and neutral expressions, in 20 pre-manifest HD gene carriers and 23 healthy controls. On the basis of the results of this initial study went on to look at amygdala dependent cognitive performance in 79 Huntington's disease patients from a cross-section of disease stages (pre-manifest to late disease) and 26 healthy controls, using a validated theory of mind task: “the Reading the Mind in the Eyes Test” which has been previously been shown to be amygdala dependent.

**Results:**

Psychophysiological interaction analysis identified reduced connectivity between the left amygdala and right fusiform facial area in pre-manifest HD gene carriers compared to controls when viewing angry compared to neutral faces. Change in PPI connectivity scores correlated with predicted time to disease onset (*r*=0.45, *p*<0.05). Furthermore, performance on the “Reading the Mind in the Eyes Test” correlated negatively with proximity to disease onset and became progressively worse with each stage of disease.

**Conclusion:**

Abnormalities in the neural networks underlying social cognition and emotional processing can be detected prior to clinical diagnosis in Huntington's disease. Connectivity between the amygdala and other brain regions is impacted by the disease process in pre-manifest HD and may therefore be a useful way of identifying participants who are approaching a clinical diagnosis. Furthermore, the “Reading the Mind in the Eyes Test” is a surrogate measure of amygdala function that is clinically useful across the entire cross-section of disease stages in HD.

## Highlights

• Reduced connectivity between the left amygdala and right FFA during emotional processing in pre-HD.
• Amygdala dependent theory of mind performance correlated with estimated time to onset in pre-HD.
• Theory of mind performance correlated with PPI connectivity in a small pre-HD sub-group.

## 1 Introduction

Huntington's disease (HD) is an incurable, progressive, neurodegenerative disorder characterised clinically by a triad of motor, cognitive and psychiatric problems (Bates et al., 2002) which is caused by an expanded cytosine–adenine–guanine (CAG) repeat in exon 1 of the huntingtin gene. Neuropathological changes can be detected decades before clinical signs emerge (Aylward et al., 2004; Paulsen, 2010) beginning in the striatum and progressing to widespread brain atrophy (Vonsattel et al., 2008). Although HD is diagnosed based on the presence of unequivocal motor abnormalities, cognitive abnormalities can be detected in most gene carriers prior to this point.

The cognitive profile of manifest HD includes deficits in executive function, emotional processing and memory (Ho et al., 2003; Henley et al., 2012; Tabrizi et al., 2013; Holl et al., 2013; Nicoll et al., 2014; Georgiou-Karistianis et al., 2013, 2014; Johnson et al., 2007; Stout et al., 2011; Begeti et al., 2013). In the prodromal phase the impairment is more subtle but abnormalities in psychomotor processing speed, verbal fluency and the recognition of negative emotions are common (Tabrizi et al., 2013, 2012; Begeti et al., 2013). The direct functional implications of these cognitive changes are still unclear (Kirkwood et al., 2002; Duff et al., 2010; Van Liew et al., 2013) but, reduced occupational performance and difficulty managing finances can be seen in pre-manifest HD gene carriers (pre-HD) who are approaching diagnosis (Beglinger et al., 2010). Furthermore, changes in personality and difficulties with social interaction are key features of early HD. One explanation for these occupational and social problems is an emerging impairment in emotional oversight e.g. accurately identifying, interpreting and responding to the emotions and intentions of others all of which are necessary for maintaining interpersonal interactions and socially appropriate behaviour.

Multiple studies have shown that HD patients are impaired on emotion recognition tasks (Johnson et al., 2007; Sprengelmeyer et al., 2006, 1996; Milders et al., 2003; Henley et al., 2008; Gray et al., 1997; Hennenlotter et al., 2004; Montagne et al., 2006; Wang et al., 2003; Hayes et al., 2007; Mitchell et al., 2005). A recent systematic review of the literature demonstrated that anger recognition is the most consistently reported impairment, closely followed by disgust and fear recognition (Henley et al., 2012) in manifest disease. While in PMGC's, selective impairments in disgust recognition have been found (Sprengelmeyer et al., 2006; Gray et al., 1997; Hennenlotter et al., 2004) and a relationship between anger recognition and proximity to estimated time of disease onset has been reported (Johnson et al., 2007). However, some studies argue that there is a more generalised impairment encompassing all negative emotions (Johnson et al., 2007), with change in negative emotion recognition over a three year period having positive predictive value for identifying PMGC's who reached a clinical diagnosis during that time (Tabrizi et al., 2013). As such, emotion recognition may be a useful marker of very early disease related changes in HD.

The underlying neural substrates of emotion recognition deficits in HD have typically been established using correlative structural MRI studies (Tabrizi et al., 2013; Johnson et al., 2007; Henley et al., 2008; Kipps et al., 2007). Such studies have identified correlations between tissue degeneration in the striatum associated with impaired recognition of surprise, disgust, anger and fear (Henley et al., 2008); between the cerebellum (Scharmuller et al., 2013) and anger recognition and between the anterior insula and disgust recognition in both manifest (Henley et al., 2008; Hennenlotter et al., 2004; Kipps et al., 2007) and pre-manifest patients (Hennenlotter et al., 2004). It has been argued however, that disease-related behavioural changes in HD are more likely to relate to disruption of the complex interactions between multiple brain circuits rather than as a result of distinct regional tissue degeneration (Paulsen, 2009) which cannot be measured on structural MRI.

Functional MRI has been used to interrogate emotional processing in PMGC's in a small number of studies which look at changes in Bold Oxygen Level Dependent (BOLD) response in brain regions during emotional processing. This approach can therefore detect disease related changes earlier than the classic approach. Dogan et al. (2013) asked PMGC's to complete an emotion recognition task whilst undergoing fMRI and reported that negative stimuli evoked decreased activation in the amygdala, hippocampus, striatum, insula, cingulate and prefrontal cortices, as well as in sensorimotor, temporal and visual areas. Other studies measure implicit emotion perception to reduce the confounding effects of performance on BOLD response, by asking participants to perform a distracter task such as a gender decision task. Hennenlotter and colleagues (Hennenlotter et al., 2004) looked at neural activation to grey scale pictures of faces displaying either disgusted, surprised or neutral expressions in PMGC's. BOLD response was reported to be lower than controls in the left dorsal (intermediate) anterior insula/opercular region and left putamen during disgust (relative to neutral) processing. However, Novak and colleagues found activation differences in a widely distributed network of brain regions involved including prefrontal, partietal and cingulate cortices during disgust, anger and happiness processing which was not restricted to any particular emotional expression or emotion valence (Novak et al., 2012).

ToM refers to an individual's ability to understand the presence of beliefs, feelings, intentions and interests in other people that can differ from their own and from reality (Baird and Astington, 2004). The ability to attribute mental states to others is likely to have a central role on human social interaction as it allows us to predict the behaviour of others. Furthermore, affective ToM and emotion recognition have been shown to activate overlapping brain regions, namely the inferior frontal gyrus, the superior temporal sulcus, the temporal pole and the amygdala (Mier et al., 2010) Despite this, ToM is an area of research that has received relatively little attention in HD. Changes in empathy have been found in patients with manifest HD demonstrated by their impaired interpretation of humorous cartoons and story vignettes (Snowden et al., 2003). Further abnormalities have been shown in similar populations of HD patients on ToM tasks such as the “Reading the Mind in the Eyes Task” (RMET) and the faux pas task (Eddy et al., 2012, 2014; Allain et al., 2011) with deficits in ToM found to relate to executive functioning (Allain et al., 2011; Brune et al., 2011) however, to our knowledge however, ToM has not been studied in PMGC's. In this study the RMET was used as a surrogate clinical measure of amygdala function on the basis of previous studies (Adolphs et al., 2002), rather than to interrogate ToM in HD.

In the current study we used an implicit emotional processing task to look for differences in neural activation between PMGC's and healthy controls when viewing grey scale pictures of angry and neutral faces. Unlike previous studies, the pictures of faces were contrasted with pictures of buildings and participants were asked to respond indicating whether they saw a face or a house on the screen. Houses were used as a contrast in this task to increase the power to functionally detect differences in BOLD response during the processing of angry but not neutral faces and not to mask the effect of brain regions which have been previously shown to be activated, non-discriminately by all facial emotions (Fitzgerald et al., 2006).

Connectivity analysis of the results indicated that abnormalities in the way that activity in the amygdala covaries with other brain regions during emotional processing may be an early disease related marker in PMGC's. To identify whether this could be measured clinically, a validated theory of mind test (ToM) which has previously been shown to impaired in patients with lesions to the amygdala (Stone et al., 2003): the Reading the Mind in the Eyes Test (RMET) (Baron-Cohen et al., 2001), was used in a population of PMGC's (11 of whom also underwent the fMRI study) and extended to a population of manifest patient from all different stages of the disease.

The combination of the two experiments provides a comprehensive assessment of amygdala related emotional processing in HD from the earliest pre-manifest stage of the disease through to advanced HD. On the basis of the existing literature we initially predicted that PMGC's would have decreased activation in and connectivity in a wide network of brain regions compared to controls when processing emotional stimuli during fMRI. Following the imaging study we then went on to hypothesise that abnormalities in ToM performance would increase progressively at more advanced stages of the disease in HD.

## 2 Methods, materials and results

### 2.1 Participants

All participants were recruited from the multidisciplinary Huntington's disease service clinic at the John Van Geest Centre for Brain Repair, UK. Control subjects were recruited through links with the clinic. Approval for this study was granted by the Local Regional Ethics Committee and Addenbrooke's hospital R&D department. Informed consent was taken from participants.

Two cohorts of participants were recruited:

1. 20 Pre-manifest HD gene carriers (PMGC) (10 males, average age=45.8 years S.D=11.16) and 23 controls (10 males, average age=42.1 years S.D.=12.04) underwent functional imaging.
2. 29 PMGC (14 males, average age=43.5 years S.D=9.5) 11 of whom were also scanned (5 males, average age=47.7 years S.D=13.2), 50 manifest patients (27 males, average age=54.4 S.D=12.1) and a further 26 different healthy controls (14 male, average age=59.0 S.D=11.7) were tested on the RMET.

Participant demographics are detailed in Tables 1 (cohort 1) and 2 (cohort 2).

### 2.2 Study 1: methods

#### 2.2.1 fMRI task

To test neural activation in response to pictures of angry faces, participants underwent functional MRI scanning. Stimuli were visible via an angled mirror positioned above their eyes reflecting images projected onto a screen at the end of the scanner bor. Responses were made using the first 2 buttons on a 4 button response box held in the participant's right hand. Participants were instructed to press button 1 to identify a house and button 2 to identify a face.

The “face” stimuli had either an angry or a neutral expression, although participants were not informed of the difference in emotional expressions and were not required to respond differently to faces of different emotions. The “face” photographs were selected from the NimStim Face Stimulus Set (www.macbrain.org) and the Karolinska directed emotional faces (KDEF); they were chosen on the basis of independent emotional ratings. Participants were presented with each expression 32 times giving a total of 64 pictures of faces from 33 separate actors. The gender and identify of faces was fully randomised. 320 pictorial stimuli were presented in total with the remaining 256 pictures made up of 20 different houses. The two performance effects of interest were infrequent (face) vs. frequent (house) stimuli and angry vs. neutral face stimuli.

Pictures were presented in a predefined pseudo-randomised order followed by a low contrast central cross. Each stimulus was presented for 750 ms, with an inter-trial interval of 750 ms to encourage participants to respond quickly and instinctively to the pictures and to reduce awareness of the overt emotional content of the face stimulus. The total experiment lasted for 9 min 20 s.

#### 2.2.2 fMRI data acquisition

Patients were scanned at the MRC Cognition and Brain Sciences Unit, Cambridge, using a 3T Siemens TIM Trio MRI scanner. During the task 290 T2-weighted echo-planar images depicting BOLD signal were acquired, the first 10 of which were discarded to avoid T1-equilibrium effects. Each image consisted of 32 slices of 3 mm thickness with a 1 mm interslice gap and an in-plane resolution of 3×3 mm^2^. The repetition time (TR) was 2 s with an echo time (TE) of 30 s. Slices were angled away from the orbits to avoid signal dropout due to magnetic susceptibility inhomogeneity. Stimuli were presented on a screen with a resolution of 1024×768 pixels which was visualised using a mirror positioned within the scanner at a viewing distance of 90 mm.

#### 2.2.3 Data analysis

##### 2.2.3.1 Behavioural data

Behavioural performance was evaluated using two outcome measures: accuracy (percentage of pictures correctly classified) and mean latency to response for both angry and neutral faces. No accuracy measures were taken for the “house” stimuli. Multivariate analysis of variance (MANOVA) was used to compare performance between gene carriers and controls and a repeated measures analysis of variance (rm-ANOVA) was used to compare performance between emotional conditions.

##### 2.2.3.2 Imaging data preprocessing

MRI data were processed using SPM8 (www.fil.ion.ucl.ac.uk/spm). Functional MRI data were converted from DICOM to NIFTII images, spatially realigned to the first image, and corrected for acquisition delay with references to the middle slice. There was no exclusion of subjects. All subjects that had finished the EGNG task were included in the analysis. To determine whether the preHD group had greater movements during image acquisition than controls', a measure of displacement was obtained for each subject using the motion correction parameters from SPM realignment function. We took the translation and rotation measurements in *x*, *y*, and *z* co-ordinates between each volume and then calculated the root mean square of the three translations and the three rotations. We then summed the translation and rotation measures across all the volumes to give indexes of the total displacement for each subject. There was no significant effect of group (pre-HD or control) on total amount of translation (*F*(1,42)=3.24, *p*=0.08) or rotation (*F*(1,42)=1.64, *p*=0.21) during scanning. The mean fMRI and MP-RAGE images were coregistered using mutual information, and the MP-RAGE image was segmented and normalised to the Montreal Neurological Institute (MNI) T1 template by linear and non-linear deformations. The normalisation parameters were applied to all spatiotemporally realigned functional images, and normalised images were resampled to 2×2×2 mm^3^ before smoothing with an isotropic Gaussian kernel with full-width half-maximum of 8 mm.

##### 2.2.3.3 fMRI data analysis

A first level general linear model (GLM) included three epoch regressors (angry faces, neutral faces, and houses) for trials with correct responses. Additional regressors representing trials with incorrect or omitted responses and six rigid-body motion correction parameters were included as nuisance covariates. Regressors were convolved with a canonical hemodynamic response function, and the data were high-pass filtered with a frequency cutoff at 128 s.

To assess brain activity associated with angry processing, first-level contrast images were generated for angry vs. neutral faces and these were entered into a second-level analysis to test for averaged effects across participants and group effects between PMGC and controls.

##### 2.2.3.4 Psychophysiological interactions for brain connectivity analysis

A PPI analysis was performed to examine the functional connectivity between the amygdala and other potential brain regions during emotional processing (Passamonti et al., 2008; Friston et al., 1997). The PPI analysis tested how physiological connectivity between a source region at amygdala and the rest of the brain varied with the psychological context (i.e., angry vs. neutral faces).

Our primary interest is the angry vs. neutral faces comparison in the connectivity analysis. A second contrast, angry faces vs. houses was used to increase the power to functionally detect the amygdala, because neutral faces have also been shown to active the amygdala (Fitzgerald et al., 2006; Wright and Liu, 2006). Two further contrasts (faces vs. houses and houses vs. faces) were conducted as a sanity check, ensuring that our task activates the functionally specific regions. Note that previous studies showed that the comparison between angry faces to houses increased the power to detect the amygdala (Passamonti et al., 2008). Although the task has been shown to active the amygdala in this and previous studies, the cluster extends beyond amygdala (see Fig. S1, supplementary data). Therefore it is not straightforward to use the fMRI results as a localizer. Here we used the same approach as in our previous study (Passamonti et al., 2008) where the contrast angry faces vs. houses was used to find the peak voxel in the amygdala (−24, −4, −16), and defined a 10-mm sphere around this peak coordinate. Our previous study showed that this approach gave similar result as defining a subject-specific ROIs (Passamonti et al., 2008).

For each participant, we computed the first eigenvariate of the BOLD time courses from all voxels in the left amygdala ROI and derived the “neural signal” of the source region by deconvolving the hemodynamic response function. The psychophysiological interaction regressor was calculated as the product of the deconvolved time course and a vector coding for the psychological variable (1 for angry faces, −1 for neutral faces). Participant specific PPI models included the psychological (angry faces vs. neutral faces), physiological (left amygdala signal), and psychophysiological variables and were re-convolved by the canonical hemodynamic response function. Six motion correction parameters were included as nuisance covariates. First-level contrast images were generated for PPIs and were entered into second level GLM analysis for contrasts of interest.

### 2.3 Study 1: results

#### 2.3.1 Demographics

Patient demographics and clinical characteristics are shown in detail in Table 2. PMCG's were well matched with controls for age. As predicted the PMGC “close” and “far” groups differed from each other in terms of estimated time to onset (*F*(1,19)=26.68, *p*<0.0001), disease burden score (*F*(1,19)=17.02, *p*<0.001) and UHDRS score (*F*(1,19)=6.25, *p*<0.05).

#### 2.3.2 Behavioural results

Patients responded to pictures of faces as quickly (*F*(3,47)=0.925, *p*=0.44) and accurately (*F*(3,47)=2.08, *p*=0.13) as controls. The angry/neutral contrast was an implicit feature of the task design, however there was no significant effect of emotion on accuracy (*F*(3,40)=1.803, *p*=0.162) or latency (*F*(3,40)=0.783, *p*=0.511).

#### 2.3.3 Connectivity analysis

**Fig. 1:**
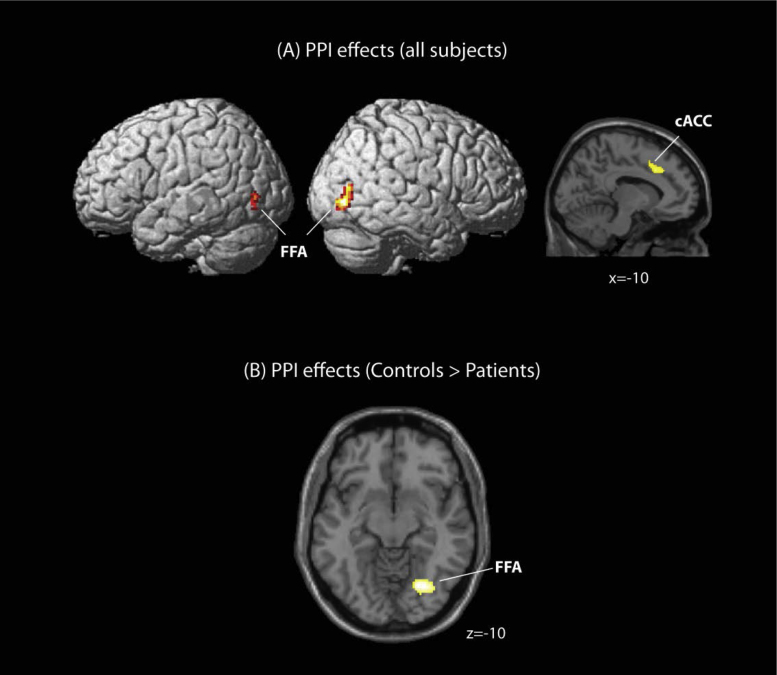
PPI GLM statistical parametrical maps. (A) Positive PPI effects originating from the amygdala during exposure to ‘angry faces’ relative to ‘neutral faces’ for all participants at a liberal threshold (*p*<0.001 uncorrected, cluster size 50 voxels or more). (B) Decreased PPI connectivity in PMGC's during exposure to ‘angry faces’ relative to ‘neutral faces’ (*p*<0.05, cluster level corrected).

Details on the brain regions activated during the task have been reported in supplementary Fig. S1. we defined the left amygdala as the seed region (10 mM sphere centred at −24, −4, −16) from an “angry faces” minus “houses” contrast for PPI analysis (supplementary Fig. S2, also see PPI connectivity analysis in Section 2). neural activity in the seed region was found to covary positively with the bilateral fusiform facial area (FFA) (left: −32, −78, −8; right: 38, −78, −4) and caudal anterior cingulate (left: −10, 20, 36; right: 16, 8, 34) across all participants during exposure to angry faces (Fig. 1A). Differences were detected in the extent of PPI activity between the left amygdala and the right FFA (30, −70, −10) (Fig. 1B) with PMGC exhibiting reduced connectivity compared to controls, corrected for multiple comparison at the cluster level (FDR corrected *p*<0.05).

**Fig. 2:**
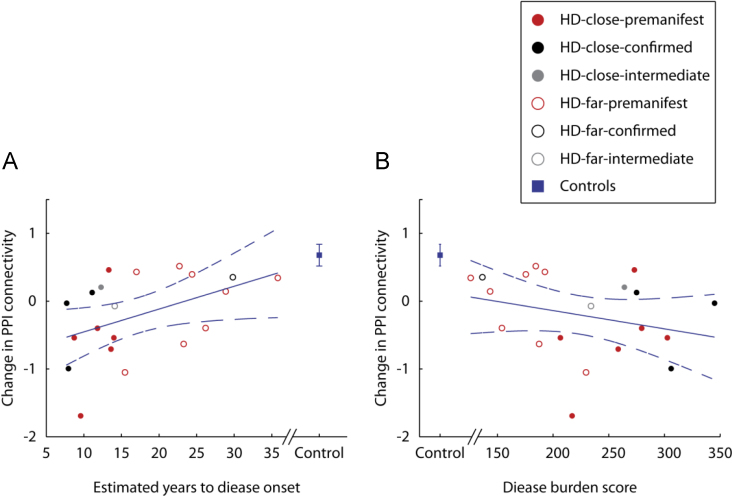
Correlation between PPI connectivity originating from the amygdala to the right fusiform facial area and (A) estimated years to disease onset and (B) disease burden score in PMGC's. Participants have be divided into “close” to and “far” from onset groups and further subdivided into pre-manifest, intermediate and confirmed groups to visually represent those gene carriers who have received (confirmed) or are anticipated to imminently receive a diagnosis of HD (intermediate) since scanning. Error bars show the standard errors across all healthy controls. The regression line (solid) and the 95% confidence intervals (dashed) are shown.

PPI connectivity between the left amygdala and the right FFA correlated significantly with estimated years to disease onset (*r*=0.45, *p*<0.05) but not disease burden score (*r*=−0.28, *p*=0.24) (Fig. 2). Change in PPI connectivity scores did not correlate significantly with age for controls (*r*=−0.04, *p*=0.86) but there was a significant relationship in PMGC (*r*=−0.54, *p*<0.01).

### 2.4 Study 2: methods

#### 2.4.1 Background assessments

Pre-morbid verbal IQ was estimated using the National Adult Reading Test (NART) (Nelson, 1982). Depressive symptomatology was evaluated using the Beck Depression Inventory revised (BDI) (Beck et al., 1961), global cognitive function was measured using the Mini Mental State Exam (MMSE) (Folstein et al., 1975) and verbal fluency was measured to provide a small insight into executive dysfunction (Henry et al., 2005).

Motor impairment and daily functioning were assessed by an experienced neurologist using the UHDRS (Huntington Study Group, 1996) motor, total functional assessment, total functional capacity and independence score subscales for all HD gene carriers. Gene carriers with a UHDRS score of ≤5 were classified as PMCG's. Manifest patients were staged according to previously published criteria based on the Total Functional Capacity (TFC) score: scores of between 11 and 13 were classified as early disease, between 7 and 10 as moderate disease and scores of 6 and less as late disease (Begeti et al., 2013).

#### 2.4.2 Social cognition task

The Reading the Mind in the Eyes Task (RMET) (Baron-Cohen et al., 2001) is a measure of affective ToM. Participants are presented with a picture showing the eye region in isolation from the rest of the face. The participant is required to say which of 4 emotional/mental state words positioned around the picture best captures the thoughts or feelings portrayed in the eyes. Total number of correct responses was recorded.

#### 2.4.3 Data analysis

Levene's test for Equality of Error Variances confirmed homogeneity of variance therefore between-group differences were examined using analysis of variance (ANOVA) with post-hoc comparison by *t*-tests with Bonferroni correction for multiple comparisons. However, when tested at the whole group level using a Shapiro–Wilk test (*p*=0.18), performance on the RMET was not normally distributed therefore performance was correlated with clinical markers of disease progression (UHDRS motor score and disease burden score) and daily functioning (TFA) using a Spearman's Rho non-parametric correlation. Furthermore, the data was normalised and linear regression analyses were used to quantify the strength of the relationship between RMET and UHDRS, DBS, and NART. The following variables were not included because of their relationship with other variables in the regression analyses: age because it was strongly associated with DBS, verbal fluency because it was associated with NART and BDI because it was associated with UHDRS. All analyses were performed on Predictive Analytic SoftWare (PASW) Statistics, version 21.

### 2.5 Study 2: results

#### 2.5.1 Demographics

Participant demographics and clinical characteristics are shown in detail in Table 1. In general the manifest patients were well matched with controls for age, NART and BDI however, PMGC were significantly younger than controls (*F*(5,97)=5.50, *p*<0.001) and late stage patients had a significantly lower NART than controls (*F*(5,96)=2.57, *p*<0.05). As anticipated, manifest patients were found to have a higher UHDRS (*F*(4,74)=65.56, *p*<0.001), lower total functional assessment (FA) scores (*F*(4,74)=156.5, *p*<0.001), lower verbal fluency score than PMGC's (*F*(3,72)=10.00, *p*<0.01) and lower MMSE scores (*F*(5,92)=12.37, *p*<0.001) compared to PMGC's. It should also be noted that gender distribution in the early HD group was uneven with more men than women and may therefore effect the analysis.

#### 2.5.2 Manifest HD

**Fig. 3:**
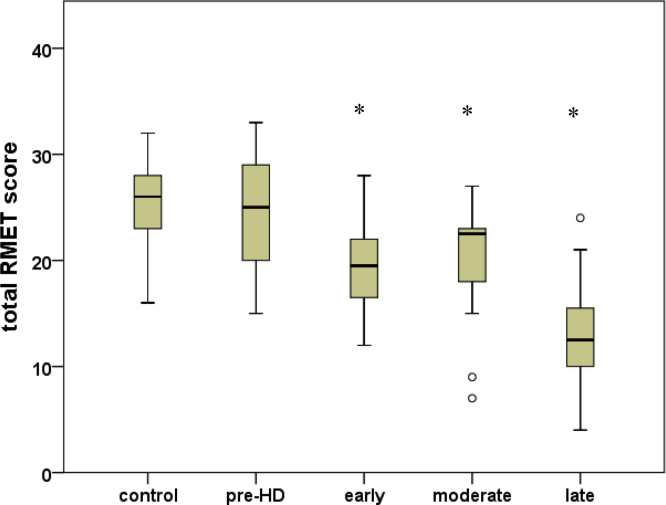
Behavioural performance on the Reading the Mind in the Eyes task stratified by disease stage for all HD participants. ⁎ indicates a significant difference compared to controls at the *p*=0.05 level.

Total number of correct responses on the RMET task deteriorated significantly with disease stage (*F*(7,65)=9.377, *p*<0.001) (Fig. 3). Post hoc investigation revealed that PMCG's were the only group not to differ from controls (*p*=1.0).

**Fig. 4:**
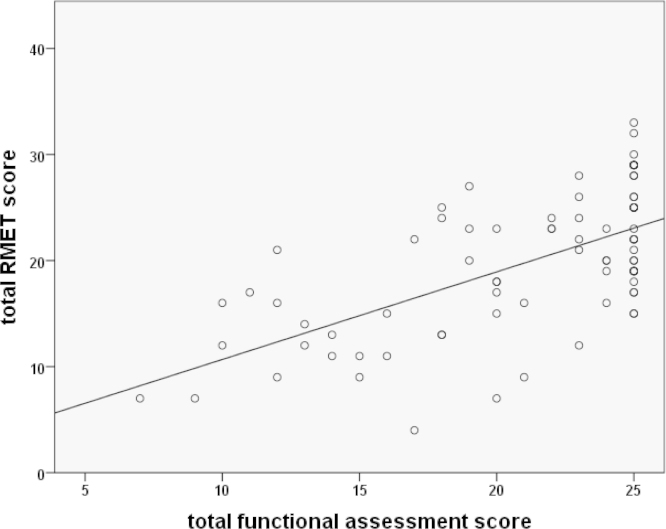
Performance on the Reading the Mind in the Eyes Tasks correlated with total functional assessment score from the UHDRS.

Performance on the RMET task correlated with motor impairment (*r*^2^=−0.71, *p*<0.001), disease burden score (*r*^2^= −0.69, *p*<0.001) and the UHDRS functional assessment (FA) score (*r*^2^=0.60, *p*<0.01), Fig. 4. Linear regression analyses identified that RMET performance related to UHDRS (*t*=−4.0, *p*<0.001), NART (*t*=3.64, *p*<0.001) and DBS (*t*=−2.17, *p*<0.05).

#### 2.5.3 Pre-manifest HD gene carriers

**Fig. 5:**
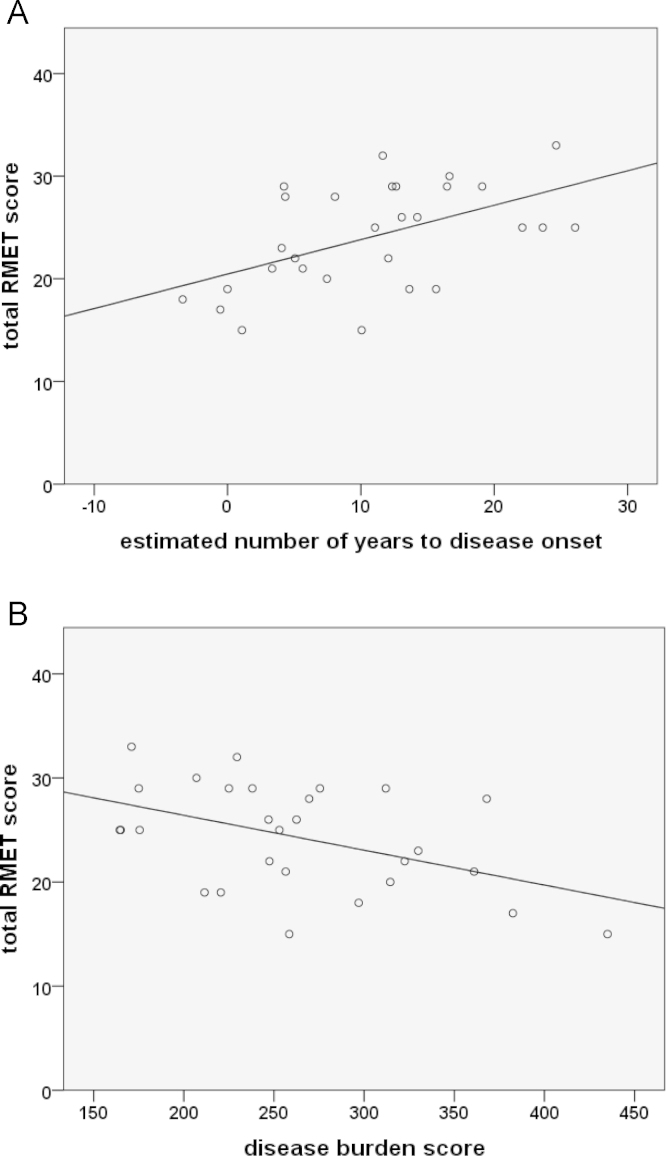
Correlation between performance on the Reading the Mind in the Eyes task and (A) the estimated number of years to disease onset and (B) the disease burden score in PMGC's.

Performance on the RMET task correlated with the number of estimated years to disease onset (*r*^2^=0.52, *p*<0.005) and disease burden score (*r*^2^=−0.41, *p*<0.05) (Fig. 5) in PMGC's with performance deteriorating as the time to disease onset shortened. However, on all other measures PMGC's were equivalent to controls.

#### 2.5.4 PPI connectivity and performance on the RMET

**Fig. 6:**
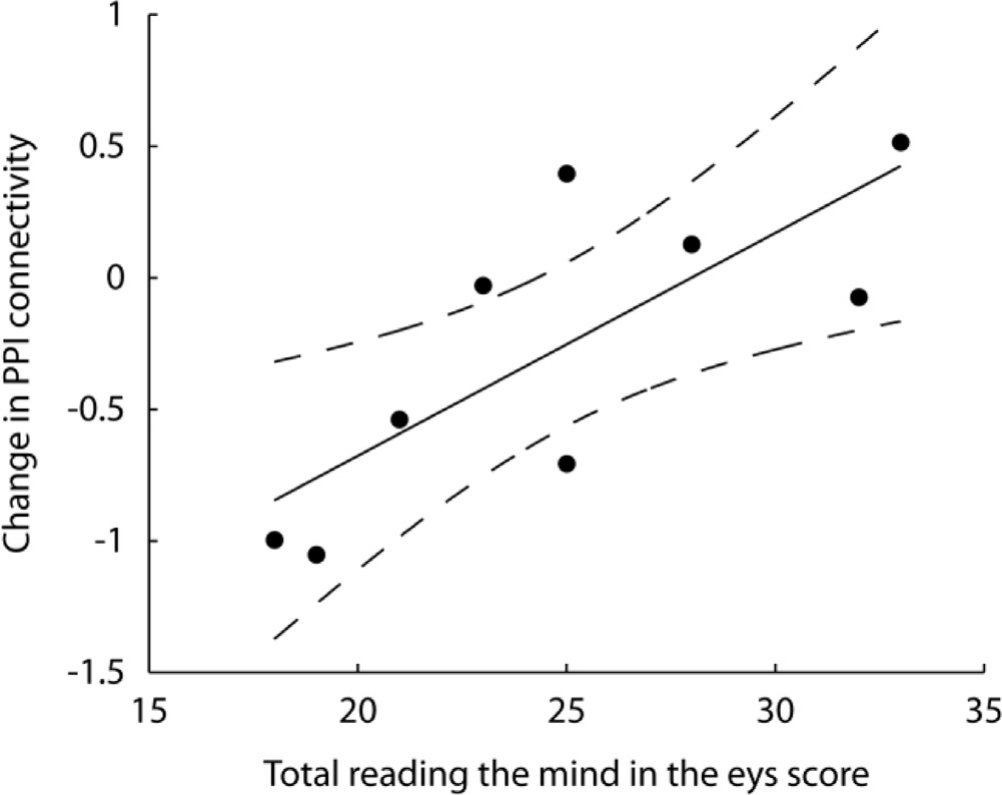
Correlation between PPI connectivity between the amygdala and the right fusiform facial area and performance on the Reading the Mind in the Eyes Task in PMGC's. The regression line (solid) and the 95% confidence intervals (dashed) are shown.

Performance on the RMET and change in PPI connectivity between the left amydgala and rFFA was not significantly correlated (Fig. 6; *r*=0.38, *p*=0.25). However, when the data from the 2 PMGC's who completed the two studies greater than 1 year apart was removed, performance on the RMET significantly correlated with change in PPI connectivity (*r*=0.77, *p*<0.05). Although caution should be expressed when interpreting these data due to the small sample size they provide relevant exploratory insights.

## 3 Discussion

The combined results of these studies suggest that the amygdala is affected early in the course of HD; even from the late pre-manifest stage. Specifically, connectivity between the left amygdala and the right FFA reduces in line with estimated time to disease onset during emotional processing; the magnitude of this effect relates to proximity to disease onset. Additionally, performance on the RMET, a task known to activate the amygdala (Stone et al., 2003), in PMGC's also correlated with estimated proximity to disease onset and deteriorated further with every cross-sectional disease stage. Finally, PPI connectivity and RMET performance correlated highly in the small subgroup of PMGC's who completed both studies within a 1 year window.

To our knowledge this is the first study to look at the influence of disease stage on social cognition performance and the first to report a relationship with predicted time to disease onset in PMGC's. Consistent with the work of others, the RMET was found to be a sensitive tool capable of detecting deficits in social cognition in HD (Eddy et al., 2012; Allain et al., 2011). In addition however, we were able to demonstrate that performance on the RMET was influenced by the length of exposure to the pathological effects of the CAG expansion (DBS), motor symptomatology (UHDRS) and cognitive reserve (NART). All of which support the use of the RMET as a clinical outcome measure in HD.

Successful completion of the RMET is heavily reliant upon activation of the amygdala (Stone et al., 2003); a region that has been implicated in face (Rolls, 1992) and facial emotion processing (Harris et al., 2014), specifically during recognition of fearful faces (Adolphs et al., 1994). Analysis of functional MRI data collected in this study found reduced functional connectivity between the left amygdala and rFFA in PMGC's compared to healthy controls when exposed to pictures of angry faces. Reduced functional connectivity has previously been shown in PMGC's during tasks of working memory (Georgiou-Karistianis et al., 2013; Poudel et al., 2015), planning (Unschuld et al., 2013) and motor performance (Unschuld et al., 2012). However, to the best of our knowledge, this is the first study to look at functional connectivity during emotional processing in such a group. Of relevance to this is a recent meta-analysis looking at regions of degeneration in PMGC's which identified the amygdala as a region commonly reported as vulnerable to HD neuropathology (Dogan et al., 2013). In addition the amygdala has also been linked to subjective fear responses in manifest HD (Eddy et al., 2011) and the recognition of emotions such as disgust and happiness (Hayes et al., 2007; Kipps et al., 2007) in PMGC's; while in rodent models of HD neuropathology in the amygdala has been shown to be associated with social and emotional memory (Ciamei and Morton, 2008; Faure et al., 2013), and motivational processes (Faure et al., 2011). Consistent with this literature the current study provides evidence of amygdala dysfunction in HD beginning prior to the onset of overt clinical features.

Both tasks used in this study involve some element of social cognition and are both reliant upon amygdala function thereby implicating the amygdala in the process of interpreting social meaning (Adolphs et al., 2002, 1994) rather than in the identification of specific emotions. Additional support for this theory comes from emerging evidence from the wider literature that suggests that the amygdala is responsible for detecting perceptual salience and biological relevance in facial emotions (Adolphs, 2008; Sander et al., 2005). Furthermore, a recent study of neurosurgical patients with chronically implanted depth electrodes in the amygdalae demonstrated that there are neurons in this structure that code specifically for both fearful and happy faces (Wang et al., 2014).

The complementary evidence from both the RMET and fMRI studies presented here, especially the involvement of the amygdala in both tasks support the rationale that both social cognition and emotional processing in HD share a common neural pathway. Moreover, it has previously been reported that impairments in the recognition of fear following amygdala damage are a result of an inability to fixate on the eye region of the face (Adolphs et al., 2005), therefore we propose that the wider emotional processing/social cognition problems seen in HD are caused by a similar impairment: a shift in attention away from the eye region of the face during emotional processing, mediated by neuronal dysfunction in the amygdala. Further work is needed to directly test this theory and the RMET may be a useful and reliable way of measuring the functional integrity of this pathway. It may also be useful, in combination with measures such as statistical estimates of disease burden and the presence of subtle motor signs, as a way of identifying PMGC's at risk of phenoconverting.

It is noteworthy that the PPI connectivity correlated with predicted time to disease onset but not DBS which initially appears surprising however, may be explained by the differences between the two models. Predicted time to disease onset was calculated using the Langbehn et al. (2004) survival analysis equation in this study. This model deals exclusively with the pre-manifest stage of HD and estimates the age at which a gene carrier will meet the criteria for clinical diagnosis. Age at diagnosis has been shown to be highly influenced by the CAG length (Langbehn et al., 2010). However, CAG length has less of an effect on the rate of disease progression in manifest disease. Therefore, when looking at the relationship between RMET and clinical measures of HD, the DBS (Penney et al., 1997) was used as a continuous variable that could be applied to all gene carriers (both PMGC's and manifest patients). The DBS is a linear equation that places less emphasis on the CAG repeat size and more on the age of the participant (or the time of exposure to the effects of the expansion) which provides an estimate of HD pathology. The DBS has been shown to be a good predictor of striatal pathology in post-mortem tissue (Penney et al., 1997). Based upon this, reduced PPI connectivity between the amygdala and FFA could be a useful outcome measure for monitoring proximity to disease onset but not disease progression. Therefore it may be useful future disease modifying trials in PMGC's. Conversely, the RMET is a useful tool by which to assess disease onset and progression.

The findings of this study may be useful when trying to understand the cause of the deterioration seen in daily functioning in the early stages of HD (Williams et al., 2007) although further work is needed to establish a link between the two. However, it is difficult to ascertain the impact that such a change has on the quality of life (QoL) of PMGC's and their families as the scales and assessments that are currently available to measure QoL, while useful in manifest disease, are not sensitive enough to detect change in the pre-manifest stage. Based upon the findings of this study this is an area that warrants further investigation.

There are clear limitations to the work presented here which need to be acknowledged. Due to the evolution of this project over a number of years, only a small number of PMGC's completed both the functional imaging and the RMET within a 1 year window. This reduced our power to detect between group differences, although a significant relationship was identified, but also potentially limits the generalisability of the data to a wider HD population. Further work is needed to confirm the link between amygdala connectivity and RMET in a larger cohort of PMGC's taking these points into consideration. Furthermore, emotion recognition was not tested in the PMGC's therefore we do not know whether or how reduced PPI connectivity relates to clinically measureable abnormalities in emotional processing. Also, given the potential utility of the RMET for both clinical and research endpoints, a systematic evaluation of the factors influencing ToM and emotional processing in HD is warranted. For example, executive function (Henry et al., 2006), mood (Fertuck et al., 2009) and age (Pardini and Nichelli, 2009) have all been shown to effect performance in other disorders, however, due to time restraints and the complexity of studying advanced HD patients these were not adequately considered in the current study but should be considered in future work. Furthermore, it should be noted that the PPI analyses were completed using a more liberal threshold for comparison than was used in the whole brain imaging analyses. This was because the effect seen following the PPI analysis was not of sufficient strength to withstand correction for multiple comparisons; mostly likely due to the number of participants studied. Therefore these results need to be studied further.

In summary, this work provides evidence that the amygdala and its connections are involved in the loss of high order emotional processing in HD. Both loss of effective connectivity between the amygdala and the FFA and performance on the social cognition task, correlated with time to disease onset and may be useful in identifying PMGC's who are at immediate risk of developing overt disease. In addition, social cognition performance continues to be a useful marker of emotional processing capabilities and social cognition throughout advancing disease making it a promising task for monitoring the complex cognitive changes associated with disease progression.

## Conflict of interest

None.

## Figures and Tables

**Table 1:** Demographic and clinical characteristics of all participants who completed Reading the Eyes in the Mind Task. Mean (standard deviation) reported unless otherwise stated. Between group comparisons were made using one way Analysis of Variance where appropriate.

	RMET					Combined	
	Control	PMGC	Early	Moderate	Late	PMCG	*p* Value
***N***	26	29	12	18	20	11	
**Age (yrs)**	59.0⁎⁎ (11.7)	43.5 (9.5)	54.1⁎⁎ (11.5)	52.8⁎⁎ (14.4)	56.1⁎⁎ (10.2)	47.7 (13.2)	0.001
**Gender (f:m)**	12:14	15:14	3:9	9:9	7:13	6:5	
**NART**	116.9 (5.1)	111.9 (7.1)	115.3 (7.9)	112.4 (10.6)	109.5⁎ (5.8)	111.5 (9.2)	0.34 ns
**MMSE**	29.0 (1.3)	29.2 (1.6)	28.3 (1.2)	27.5 (1.9) ⁎⁎	25.9⁎ (3.7) ⁎⁎	29.9 (0.4)	0.001
**BDI**	4.6 (3.8)	7.8 (7.9)	6.9 (6.6)	11.4 (9.3)	10.9 (5.4)	6.5 (6.0)	0.40 ns
**FAS**	ND	40.6 (14.00)	30.2 (12.5)	30.2 (15.1)	18.8 (11.3)⁎⁎	44.4 (18.8)	0.001
**UHDRS**	N/A	1.3 (2.2)	16.4⁎⁎ (8.0)	21.8⁎⁎ (10.6)	37.5⁎⁎ (14.0)	1.5 (3.0)	0.001
**TFA**	N/A	25.1 (0.4)	26.3 (1.2)	29.4⁎⁎ (1.9)	35.6⁎⁎ (2.3)	25.0 (0.0)	0.001

Data are mean (standard deviation). RMET & scanning=pre-manifest HD gene carriers who completed both studies. Abbreviations – BDI: Beck Depression Inventory, FAS: verbal fluency, MMSE: Minimental State Exam, N/A: not applicable, NART: National Adult Reading Test, ND: not done, TFA: Total Functional Assessment, UHDRS: Unified Huntington's Disease Rating Scale,

⁎ Significantly different from controls, *p*<0.05.

⁎⁎ Significantly different from pre-HD, *p*<0.05.

**Table 2:** Clinical characteristics of the PMGC's. Mean (standard deviation) is tabulated unless otherwise stated. Between group comparisons using one-way Analysis of Variance.

	PMGC close	PMGC far	Control close	Control far	*p* Value
*N*	10	10	12	11	
Gender (F:M)	3:7	5:5	5:7	8:3	
Age (years)	49.6 (11.4)	41.5 (9.8)	45.0 (13.6)	38.7 (11.1)	0.17 (ns)
CAG repeat length	42.1 (2.5)	40.3 (1.3)	n/a	n/a	0.1 (ns)
Estimated time to onset	10.5 (2.9)	23.8 (6.9)	n/a	n/a	0.001
Disease burden score	293.4 (58.6)	176.4 (37.0)	n/a	n/a	0.001
UHDRS	3.3 (2.5)	0.4 (1.0)	n/a	n/a	0.003
Diagnostic confidence score	1.0 (1.1)	0.2 (0.4)	n/a	n/a	0.039

Abbreviations – CAG: cytosine–adenine–guanine, UHDRS: Unified Huntington's Disease Rating Scale,
