# Supplementary material for: The role of the amygdala during emotional processing in Huntington's disease: From pre-manifest to late stage disease
Source: Neuropsychologia. 2015 Apr;70:80–9. doi: 10.1016/j.neuropsychologia.2015.02.017 (PMC4415907; doi:10.1016/j.neuropsychologia.2015.02.017)
Supplement: Supplementary file 1 — Supplementary Material [file mmc1.docx]

### Supplementary material


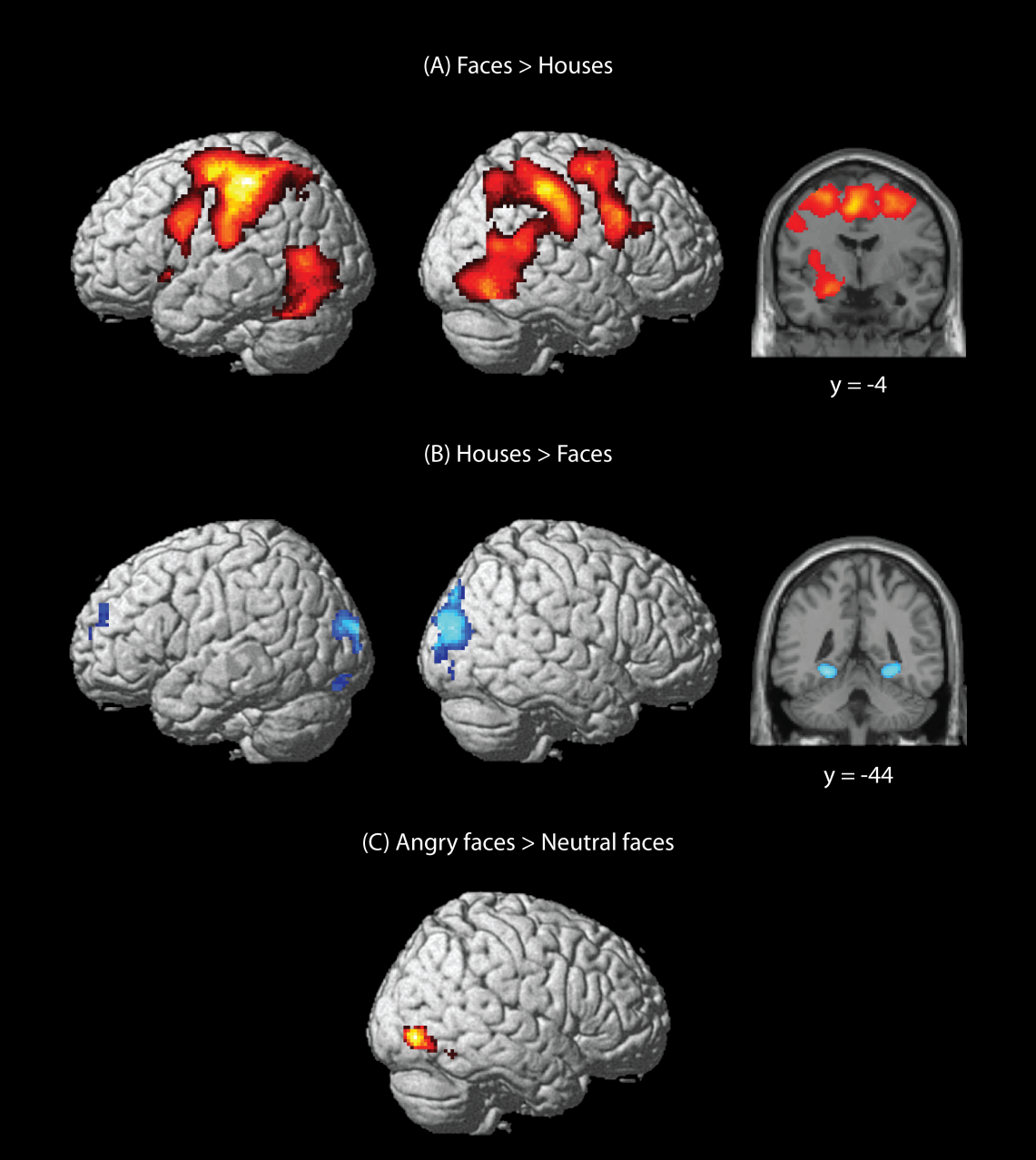


Supplementary figure S1. FMRI results across all participants (*p*<0.05 cluster level corrected). (A) Increased BOLD response to all faces versus houses stimuli in the bilateral FFA, left amygdala, sensorimotor cortex and other regions. (B) Increased BOLD response to house versus all face stimuli in the bilateral parahippocampal area (PPA), ACC, and visual cortex. (C) Increased BOLD response to angry faces versus neutral faces in the right FFA.


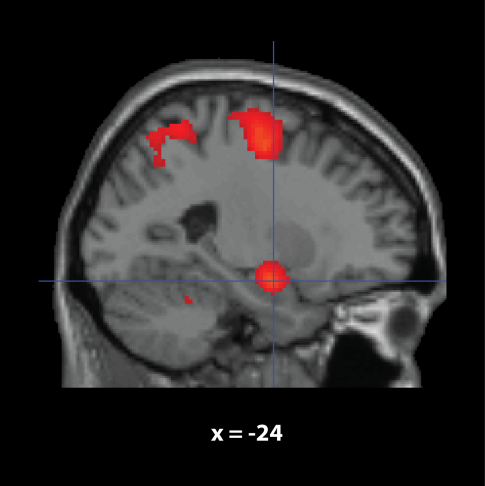


Supplementary figure S2. Source region for the PPI analysis. The left amygdala was defined as a 10 mm sphere, centered at the peak coordinate from the angry faces versus houses contrast (-24, 4, -6). The slice shown is at x = − 24 mm in MNI space.
